# Processed Transcript Insertion as a Novel Germline Mutational Mechanism in *BRCA1*-Associated Hereditary Breast Cancer

**DOI:** 10.3390/cancers17233872

**Published:** 2025-12-02

**Authors:** Anikó Bozsik, Henriett Butz, Vince Kornél Grolmusz, Petra Nagy, Tímea Pócza, Erika Tóth, Erzsébet Csernák, Attila Patócs, János Papp

**Affiliations:** 1 Department of Molecular Genetics and National Tumour Biology Laboratory, National Institute of Oncology, Comprehensive Cancer Center, 1122 Budapest, Hungary; butz.henriett@oncol.hu (H.B.); grolmusz.vince@oncol.hu (V.K.G.); nagy.petra@oncol.hu (P.N.); pocza.timea@oncol.hu (T.P.); patocs.attila@oncol.hu (A.P.); papp.janos@oncol.hu (J.P.); 2 HUN-REN-OOI-TTK-HCEMM Oncogenomics Research Group, 1054 Budapest, Hungary; 3 Department of Laboratory Medicine, Semmelweis University, 1122 Budapest, Hungary; 4 Department of Oncology Biobank, National Institute of Oncology, 1122 Budapest, Hungary; 5 MTA-OOI Lendület “Momentum” Hereditary Cancers Systems Biology Research Group, 1122 Budapest, Hungary; 6 Department of Molecular Pathology, National Institute of Oncology, Comprehensive Cancer Center, 1122 Budapest, Hungary; dr.toth.erika@oncol.hu (E.T.); csernak.erzsebet@oncol.hu (E.C.)

**Keywords:** transposon insertion, germline pathogenic variant, structural variation, hereditary breast cancer, target-primed reverse transcription

## Abstract

**Background/Objectives**: Germline *BRCA1* mutations account for ~15–20% of hereditary breast and ovarian cancer (HBOC) cases. While most are small sequence variants, structural rearrangements also contribute significantly to the pathogenic landscape. Conventional diagnostic workflows often miss such events, underscoring the need for comprehensive approaches. Here, we report a previously undescribed pathogenic mechanism—a transposon-mediated processed transcript insertion—expanding the mutational spectrum underlying hereditary breast cancer susceptibility. **Methods**: The studied case was discovered during our germline genotyping routine: next-generation sequencing followed by library preparation with a custom hereditary cancer panel. The identified variant was validated by orthogonal sequencing and multiplex ligation-dependent probe amplification (MLPA). RNA-level functional assays, including nonsense-mediated decay inhibition, were conducted to assess transcript stability. Constitutional origin was confirmed by analysis of multiple normal tissues, and tumor material was evaluated for loss of heterozygosity (LOH). **Results**: NGS detected a 700 bp insertion in exon 16 of *BRCA1*, corresponding to a complete processed transcript of *RPL18A*. The insertion caused a frameshift and premature stop codon, triggering degradation of the aberrant transcript. The variant was present in multiple somatic tissues, and its heritable nature was further confirmed by genotyping a first-degree relative, who was also found to carry the insertion. Tumor DNA analysis revealed strong LOH with retention of the variant allele. **Conclusions**: This study identifies, for the first time, a heritable processed transcript insertion as a pathogenic event in *BRCA1*. Such variants are undetectable by conventional diagnostic workflows lacking structural variant analysis, highlighting the importance of comprehensive approaches for accurate diagnosis and genetic counselling in hereditary cancer syndromes.

## Simple Summary

Structural variations in cancer predisposition genes significantly contribute to the pool of pathogenic variants underlying heritable cancer susceptibility. In this report, we describe and functionally characterize a novel transposon-mediated germline pathogenic insertion identified within the coding region of the *BRCA1* gene. The inserted sequence comprises the entire processed transcript of *RPL18A*, a ribosomal protein-coding gene, and may have been generated through a target-primed reverse transcription event. Robust molecular testing confirmed the heritability of the variant and provided evidence of its correlation with the observed phenotype. This is the first documented case of a germline cancer susceptibility variant arising through this unique mechanism.

## 1. Introduction

Germline genetic diagnostics of hereditary cancer syndromes are an important part of oncological care, as they significantly influence patient management [1]. Knowledge of hereditary predisposition can guide therapeutic surgical approaches and inform risk-reducing surgical options [2]. Additionally, hereditary genetic alterations can indicate targeted therapies, such as Poly (ADP-ribose) polymerase (PARP) inhibitors or immunotherapy [3]. Still, there are numerous cases in which both familial and personal clinico-characteristics suggest hereditary breast and ovarian cancer (HBOC), yet conventional germline genotyping methods do not reveal any causative variant [4]. These may be partly explained by genetic factors that are missed by routine diagnostic procedures. Some cases harbor pathogenic variants in non-conventionally genotyped regions—such as promoters, enhancers, and introns—or involve structural genetic alterations that are not easily detectable, including large insertions and inversions [5]. Special genotyping techniques and targeted analytical algorithms are required to uncover these factors. Whole-genome sequencing (WGS) is capable of identifying causative variants residing outside protein-coding sequences [6,7]. Copy number variation (CNV) analysis, calculated from normalized relative read coverage in next-generation sequencing (NGS), can detect gross deletions and duplications [8]. Structural variant (SV) software highlights large insertions and inversions [9]. Joint application of these sequence analysis methods is needed to address part of the missing heritability.

Copy number variations involving entire exons, such as deletions or duplications, account for around 10% of clinically relevant variants overall; however, this proportion can vary considerably among individual genes [10,11]. Large-scale inversions and insertions as a pathogenic source of cancer susceptibility genes are much rarer events, described only in a handful of surveys [9]. Insertions are generated through transposon movements, in which the inserted sequences are mainly small ~300 bp Alu motifs, but larger long interspersed nuclear elements (L1) and SINE-VNTR-Alu (SVA) sequences are also present at much lower frequency [8,9]. A small subset of L1 elements is the only transpositionally active motif in the human genome. These mainly replicate themselves in cis [12] but occasionally mediate transposition of other elements (short interspersed nuclear elements (SINE) and mRNAs of coding genes) in trans [13]. Transposon-mediated pseudogene—i.e., processed pseudogene—generation for various coding genes is an extremely rare event occurring in the germline on an evolutionary scale [14], but it is a more recurrent phenomenon somatically during tumor evolution [15]. Importantly, many conventional diagnostic workflows focus primarily on small sequence variants and standard copy number changes, meaning such rare structural events can remain undetected. So far, no clinically relevant processed pseudogene has been reported in germline susceptibility genes as a predisposing factor in hereditary disease syndromes. This report describes and characterizes a heritable insertion of an *RPL18A* processed transcript into the coding region of the *BRCA1* susceptibility gene, which constitutes a novel pathomechanism in hereditary tumor syndromes.

## 2. Materials and Methods

### 2.1. Germline Genotyping

Germline genetic screening was performed with eligibility criteria [1]. The patient received genetic counselling and provided informed consent for genetic testing at the Department of Molecular Genetics, National Institute of Oncology [16]. Genomic DNA was obtained from peripheral blood cells using the Gentra DNA Blood extraction Kit (QIAGEN, Hilden, Germany). Probe enrichment-based library preparation was performed by the TruSight Hereditary Cancer Panel covering 113 coding genes (#20029551, Illumina, San Diego, CA, USA) and sequenced on the NovaSeq6000 sequencer (Illumina, San Diego, CA, USA). For sequence analysis, the Illumina DRAGEN Enrichment pipeline (v.4.0.3, San Diego, CA, USA) was used with alignment to reference sequence GRCh38/hg38, evaluating small-scale sequence alterations, copy number variations (CNV) and structural variations (SV). Only regions covered by at least 20 reads were assessed. Variant data from exons and flanking introns (±50 bases relative to exon borders) were considered. A heterozygous position was accepted if the variant ratio was greater than 30%. Variant validation was done with targeted long-range PCR amplification (#RR002M, TaKaRa LA Taq^®^, Takara Bio, Shiga, Japan) and subsequent Sanger sequencing by the ABI 3130 Genetic Analyzer (ThermoFisher Scientific, Waltham, MA, USA). Primer sequences used in PCRs and Sanger sequencings are given in Supplementary Table S1. Multiplex-ligation probe amplification (MLPA) testing for copy number variation was carried out by the P002-D1 kit (MRC-Holland, Amsterdam, The Netherlands). Orthogonal NGS of tissue specimens was performed by amplicon-based library preparation method for *BRCA1/2* genes (#A32840 Oncomine™ BRCA Research Assay, Illumina) and sequenced with the Ion GeneStudio™ S5 Plus SystemNGS sequencer (ThermoFisher Scientific, Waltham, MA, USA). Variant nomenclature was given according to the relevant HGVS rules [17] (Hart et al., 2024). The study was approved by the Scientific and Research Committee of the Medical Research Council of the Ministry of Health, Hungary (ETT-TUKEB 53720-4/2019/EÜIG).

### 2.2. Tumor DNA Genotyping

Somatic DNA of tumor and normal tissues were isolated from a paraffin-embedded surgical specimen by the Max Well RSC DNA FFPE kit (cat. no. # 1450, Promega, Madison WI, USA). Genetic profiling was done both with the TruSight Hereditary Cancer Panel together with targeted Sanger sequencings followed by PCR reactions (for primer sequences, see Supplementary Table S1) and the Oncomine™ BRCA Research Assay (Illumina, San Diego, CA, USA). Loss of heterozygosity was evaluated by relative allele ratios of marker heterozygote variant rs1799966 compared to the germline. All allelic marker tests were repeated on separate DNA isolates of each specimen.

### 2.3. Transcript-Level Studies

RNA was isolated either from peripheral blood with the Tempus Spin RNA Isolation Kit (ThermoFisher Scientific, Waltham, MA, USA) or short-term–cultured peripheral blood mononuclear cells (PBMC) by the miRNeasy kit (103873, QIAGEN, Hilden, Germany), adhering to the protocols. First-strand reverse transcription was carried out by ProtoScript II Reverse Transcriptase (cat. no. E6560, New England Biolabs, Ipswich, MA, USA). cDNA primers were designed by Primer3Plus (https://www.primer3plus.com, accessed on 16 October 2023 and RT-PCR reactions were carried out with the QIAGEN Multiplex PCR Kit (cat. no. 206143, QIAGEN, Hilden, Germany). Amplification products were visualized on 1% agarose gel next to Hyper Ladder 1kb DNA sizing standard (cat. no. 33053, Bioline, London, UK) and subsequently sequenced by the conventional Sanger sequencing method on the ABI3130 Genetic Analyzer (Thermo Fisher Scientific).

### 2.4. NMD Inhibition

Cryopreserved PBMC samples were defrosted and 5 × 10^3^ cells were inoculated in 10 mL PB-MAX™ Karyotyping Medium (Gibco, Frederick, MD, USA) in a 10 mL flask and incubated in a 37 °C CO_2_ thermostat for 5 days. On the 5th day, 200 µL of 10 mg/mL puromycin (cat. no. P4512, Sigma-Aldrich, Burlington, MA, USA) was added to the medium and further incubated for 6 h prior to harvesting. A mock without puromycin was prepared in parallel for each treatment. Changes in the electrophoretic peak intensity ratios of the two alleles of rs1799966 were measured as a reliable indicator of nonsense code-mediated decay of transcripts carrying a premature termination codon.

## 3. Results

### 3.1. Discovery of a Long Insertion in BRCA1 Coding Sequence

**Figure 1:**
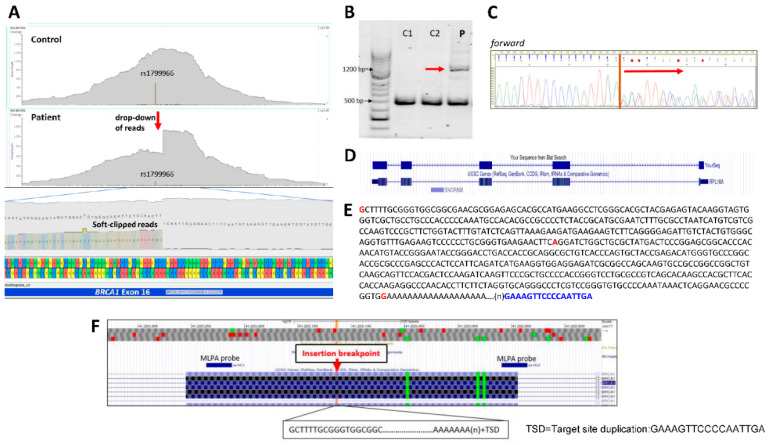
Identification and DNA-level characterization of the insertion. (**A**) Binary alignment files of the NGS reads of *BRCA1* exon16 for the patient and a control visualized in Golden Helix GenomeBrowse 3.0. The bar in the middle shows takes for heterozygote rs1799966. Strong drop-down in read depth is highlighted with red arrow. The misaligned reads are soft-clipped to show foreign sequence readout. (**B**) Agarose gel detection of amplicon covering *BRCA1* exon 16 amplified with primers B1_ex16_F and B1_ex16_R. An additional ~700 bp longer band (indicated with red arrow) was visible next to the normal product in the patient (P) relative to control (C1, C2) samples. The original original agarose gels can be found Figures S2 and S3. (**C**) Sanger sequencing electropherogram of the insertion region of the patient in forward coding direction. The vertical line signifies insertion breakpoint. Superimposed nucleotides indicate heterozygote insertion, with alleles at a roughly 1:1 ratio. (**D**) The insert sequence alignment to hg19/GRCh37 by ucsc.genome browser (https://genome-euro.ucsc.edu, accessed on 28 November 2025) BLAT application. The sequence aligned to expressed regions of *RPL18A* gene coded in chromosome 19. (**E**) Nucleotide sequence of the insertion in sense orientation to *BRCA1* code. Bases different from the ref. seq. are marked with red. Target site duplication is featured with blue. The size of the inserted sequence cannot be determined in exact base pair resolution because of the uncertain length of the poliA stretch. (**F**) Sequence surroundings of the insertion captured from ucsc.genome browser. MLPA probes of *BRCA1* P002-D1 (MRC-Holland) do not overlap insertion site. TSD: target site duplication.

In the course of our routine germline genetic testing on eligible breast cancer patients, we discovered an unusual genetic finding as a result of NGS sequencing. In a female patient, diagnosed with breast cancer at the age of 62, the structural variant (SV) analysis algorithm identified a heterozygous insertion of an unknown motif in exon 16 of the *BRCA1* gene. The read depth in this region was exceptionally high, with coverage exceeding one thousand reads. Approximately half of the hybrid reads overlapping the insertion breakpoint failed to fully align with the reference sequence. Consequently, these reads were soft-clipped at a specific nucleotide position, resulting in a marked reduction in coverage beyond that point (Figure 1A). Apart from structural variant (SV) analysis, no other annotation method was able to detect this structural alteration. The variant was too large to be fully resolved by short-read sequencing, did not impact exon copy number, and did not overlap with any MLPA probe hybridization sites (Figure 1A,F). Structural rearrangements, such as inversions or insertions, can only be detected with MLPA when their breakpoints fall within the probe’s hybridization regions. In this special case, both MLPA probes designed for exon 16 were far from the insertion breakpoint; therefore, this technique was not an applicable method for validation (Figure 1F).

To determine the sequence and length of the inserted genetic segment, we amplified *BRCA1* exon 16 using flanking intronic primers (Supplementary Table S1). This yielded an amplicon approximately 700 base pairs longer in the patient’s DNA compared to negative control samples. (Figure 1B). The larger fragment exhibited noticeably lower intensity, likely due to the inherent tendency of polymerases to preferentially amplify smaller DNA fragments. To overcome this, we designed more distal primers flanking the insertion site, encompassing approximately 7 kb of sequence, where the relative size difference of the two allelic products is not appreciable, and performed long-range PCR (LR-PCR). Sanger sequencing of the LR-PCR product with nested primers revealed the insert sequence (Figure 1C). The relative peak intensities in the sequence electropherogram superpositions was 1:1, consistent with genuine germline allelic ratios (Figure 1C). The alternative sequence was aligned to the human genome assembly hg19/GRCh37 and turned out to be a nearly perfect match to a processed transcript of the large ribosomal subunit protein RPL18A. All exons of RPL18A located on chromosome 7 were present in consecutive order, along with the 5′ and 3′ untranslated regions (UTRs) and a poly(A) tail, in sense orientation relative to the target gene (Figure 1D). With the exception of three nucleotide positions, the inserted sequence was identical to the reference sequence of the RPL18A gene (Figure 1E). One of the nucleotide differences was an addition of one base (G) at the end of the inserted sequence, which is common in linking strands by non-homologous end-joining complexes. The insertion was flanked by a duplicated 17 bp motif, GAAAGTTCCCCAATTGA, derived from *BRCA1* exon 16. The exact length of the poly-A stretch could not be determined by sequencing due to technical limitations inherent to the method. Accordingly, the correct HGVS genomic nomenclature for this variant is NC000017.11:g.43071097_43071098ins[GCTTTGCGGG…AAAA(n);43071098_43071114]. Notably, the proband also carried a heterozygous polymorphism, rs1799966 (hg19 chr17:41,223,094T>C; *BRCA1*:c.4837A>G), located 20 base pairs downstream of the insertion site. This variant is detectable in the binary alignment visualization of the patient’s NGS reads, albeit at a very low frequency (Figure 1A). This suggests that nearly all reads harboring the polymorphism—likely in phase with the insertion—were dropped during alignment. Indeed, the alternative allele of this variant was co-amplified with the insertion in all insert-specific PCR reactions, confirming that the insertion and the polymorphic variant were in cis configuration. This phasing information could be leveraged in subsequent analyses or diagnostic assays.

### 3.2. Heritability of the Variant Corroborates Its Germline Nature

**Figure 2:**
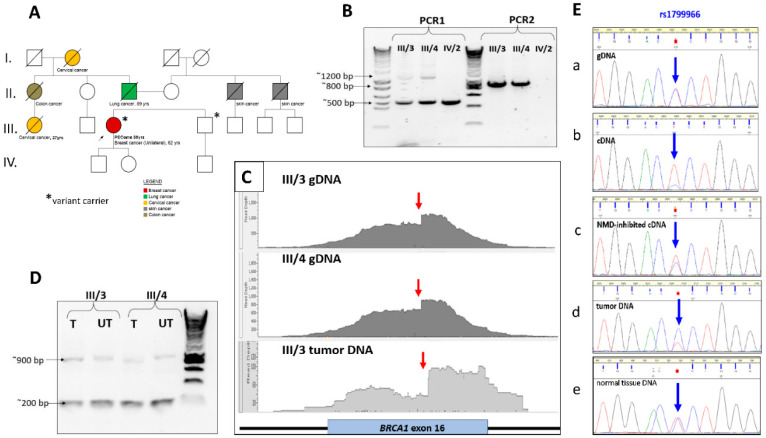
Assessing the heritability and pathogenicity of the insertion. (**A**) Pedigree of the proband. Proband is indicated with arrow. The sign * signifies the variant carriers. (**B**) Germline targeted screening of the insert in available family members. PCR1: done with primers B1_ex16_F and B1_ex16_R spanning the insertion. III/3 and III/4 had the 1200 bp extra band confirming the insertion. PCR2: done with insert-specific primers *BRCA1*_ex16_FOR and InsRPL18_REV (one of the primers binding inside the insert). III/3 and III/4 had a positive result, while IV/2 did not yield product. The original western blots can be found Figure S4. (**C**) A binary alignment map of the *BRCA1* exon 16 region, generated from next-generation sequencing (NGS) of insert-carrier DNA libraries and visualized using the Golden Helix Genome Browser. Library preparation was performed via capture-based enrichment using the Illumina Hereditary Cancer Panel. Red arrow signifies the drop-down position of read coverages. The intensity of the drop is more enhanced in the tumor. (**D**) RT-PCR reactions on NMD-inhibited and NMD-proficient transcripts of the variant carriers with primers *BRCA1*_ex16_FOR and *BRCA1*_ex16_REV. T: treated with puromycin UT: untreated. The original western blots can be found Figure S5. (**E**) Relative peak intensities of the heterozygote marker position rs1799966 in Sanger sequencing electropherograms. The sequence orientations are 5′ to 3′, corresponding to the chromosome (+) strand.

The variant carrier proband was a 62-year-old female patient diagnosed with triple negative breast cancer. Earlier, she was recognized with perivascular epithelioid cell tumor (PEComa) at the age of 58. In her pedigree, no other known family member had HBOC-related tumors (Figure 2A). Genomic DNA samples were available from two additional first-degree relatives of the proband, allowing us to perform segregation analysis. The patient’s brother (III/4) was identified as a carrier of the variant, which was confirmed using allele-specific PCR (Figure 2B) as well as by NGS method (Figure 2C). NGS analysis was validated using the proband’s DNA obtained from a second blood sample collected six months after the initial test. The read alignment profiles of both carriers were identical, showing the same drop in coverage position and intensity (Figure 2C). The patient’s daughter (IV/2) tested negative for the variant (Figure 2B). The surgical specimens of the proband were available, including tumor and adjacent normal breast tissue DNA samples, which enabled variant testing in somatic tissues. Due to the low quality and fragmentation of the extracted DNA, capture-based library preparation was not feasible; therefore, we employed an amplicon-based library preparation approach followed by orthogonal NGS sequencing. All analyzed tissues harbored the insertion, which appeared as an apparent deletion in exon 16 of *BRCA1*, since primers flanking the insertion site amplified only the wild-type allele (Supplementary Figure S1). Allelic read ratios of the heterozygote marker rs1799966 reflected well the insertion content. In all DNA specimens of the patient, these ratios showed a 1:1 balance, except in the tumor, where the alternative allele of the marker prevailed, signaling a shift towards the insert-carrier allele.

### 3.3. Transcript-Level Functional Studies Underpin Pathogenicity of the Variant

We obtained RNA from the peripheral blood of the variant carriers and transcribed it into cDNA. Taking the cDNA as a template, we designed RT-PCR reactions flanking the insert and yielded two products: one shorter, corresponding to the wild-type sequence, and one ~700 bp-longer, in accordance with the insertion (Figure 2D). Indeed, Sanger sequencing authenticated that the longer sequence contained the *RPL18A*-processed transcript code, the very same as detected in the germline, so it was represented entirely in the transcript. Accordingly, the HGVS transcript name is NM_007294.4:r.4816_4817ins[GCTTTTGCGGG……AAAA(n);GAAAGTTCCCCAATTGA]. The foreign sequence affected the coding frame of the gene incorporating false amino acids until it randomly ended up in a premature termination codon. The hypothetic truncated protein HGVS name is NP_009225.1:p.(Lys1606SerfsTer48). The nonsense code-mediated decay of the transcript was assessed with an NMD-inhibition test. Due to the large size of the insert, it could not be directly quantified in cDNA. Therefore, the rs1799966 variant, which serves as a marker for the insert, was evaluated, harnessing the knowledge that the insertion is in *cis* phase with the alternative allele of this variant. Electrophoretic ratios of rs1799966 alleles measured at the cDNA relative to gDNA showed strong diminishing of the C (alternative) allele, indicating that there is a significant mRNA-decay of the aberrant transcript (Figure 2E, panel a,b). We inhibited the NMD mechanism with puromycin on the short-term cultured PBMC of the patient and measured allelic ratios on the isolated RNA. Inhibiting NMD efficiently restored the allelic ratio experienced in gDNA (Figure 2E, panel c).

### 3.4. Loss of Heterozygosity in Tumor DNA Supports Clinical Causality

DNA isolated from the surgically excised tumor mass was investigated qualitatively as well as quantitatively for the presence of the insertion-carrier allele. Specific PCRs, where one of the two primers fell into the insert, successfully pointed out the involvement of the inserted sequence. For getting quantitative results, we used again the marker variant position rs1799966 situated in close proximity to the insertion. Peak intensity ratios of the Sanger sequence electropherogram of the amplicon targeting rs1799966 were unbiased indicators of quantitative ratios of the insert-carrying and normal alleles. We obtained that peak height of the alternative allele (C) of the marker variant, which is in *cis* with the insertion, was much higher than the reference allele (T) when compared to gDNA ratios (Figure 2E, panel d). This indicates that the majority of tumor cells predominantly harbor the inserted allele, implying loss of the normal allele and resulting in a pronounced loss of heterozygosity (LOH) in favor of the aberrant allele within the tumor genome. Capture-based NGS sequencing of the tumor DNA, although of low quality, appeared to corroborate this result. The characteristic drop in read alignments observed in gDNA was also present and appeared even more pronounced (Figure 2C, bottom). Orthogonal NGS sequencing through amplicon-based enrichment also provided precious information concerning LOH. Quantitative calculations of insert-carrying versus normal allele ratios based on read coverages were not feasible due to the substantial standard deviation of this metric. Instead, we evaluated the allelic read number ratios at the heterozygous position rs1799966, derived from a genetic segment whose amplification was unaffected by the insert. In all DNA specimens from the patient, the allelic ratio remained at 1:1, except in the tumor sample. In the tumor, the alternative allele of the marker predominated, indicating a shift toward the insert-carrying allele (Supplementary Figure S1).

## 4. Discussion

In this study, we characterized a unique germline genetic alteration of *BRCA1*, which resulted from a novel type of mechanism not reported earlier in genes associated with cancer suspicion. The variant was uncovered during routine germline genetic testing in a breast cancer patient and genotyped as a complete processed RPL18A transcript inserted into the coding region of the *BRCA1* gene. Larger insertions, typically longer than a medium read length of NGS, are not discernible by variant callers; structural variation (SV) algorithms are required to observe their breakpoints. Subsequent molecular analyses, PCR amplifications and Sanger sequencings are needed to decipher the full genetic composition of these structural variants. This peculiar alteration could be observed and correctly evaluated only by using a SV analysis algorithm applied to a captured-based enrichment sequencing. Amplicon-based sequencing misinterpreted the variant position as deletion, and an MLPA study yielded negative results, since the insertion did not affect MLPA probe hybridization targets. We could not ascertain the exact length of the polyA stretch of the insert, but this knowledge was not indispensable for declaring pathogenicity, since the inserted fragment coded for a premature termination codon well before the polyA motif. Accordingly, RNA-based functional studies confirmed that the insertion is expressed in the transcript and subject to NMD-decay. The variant pathogenicity was further reinforced by LOH of the normal allele in the tumor.

The insertion mechanism must have been a rare molecular process named target-primed reverse transcription, mediated by active L1 transposon elements in trans, copying mRNA of coding genes instead of self-copying [14,18]. In this case, the mRNA of RPL18A, an actively transcribed housekeeping gene coded on chromosome 7, was accidentally incorporated into the ectopic genetic surroundings, presumably in the course of an L1 retrotranspositional occasion [13]. The RPL18A transcript was templated by the (+) strand of chromosome 17 and inserted between nucleotide positions hg19 chr17:g.41,223,114 and 41,223,115. The insert harbors all the hallmarks of a retrotransposed sequence generated through target-primed reverse transcription. It has flanking sequences of 17 bps GAAAGTTCCCCAATTGA as target site duplication. The insertion site has the consensus 5′-TT/AAAA-3′ motif with the exception of one G base instead of an A: 5′-TT/GAAA-3′. Intriguingly, the same motif with this A>G base change was detected in several surveys as consensus [19,20,21,22]. A subset of long interspersed nuclear elements of the human genome, such as a small fraction of L1, is still capable of active transposition. These elements copy themselves at an estimated rate of one germline insertion in every 100 individuals [23]. Transposition in trans, producing processed pseudogenes, is even more scarce, or approx. 0.2-0.5% of all transposition events [14]. The insertion of an abundantly expressed gene coding for ribosomal subunit RPL18A is in agreement with the observation that highly expressed transcripts are especially likely to be templates for pseudogenes [24]. There are more than 20 partially perfect processed copies of RPL18A referred to in the human genome assemblies; some of them fall into genes, but none affect coding sequence [25]. Transposon insertions—mainly Alu sequences, but less frequently SVA and L1 elements—have already been reported in multiple surveys as germline mutations in hereditary cancer syndromes [8,26,27]. Notwithstanding, this is the first discovery of a processed transcript acting as a germline pathogenic factor in cancer predisposition genes.

No family member of the proband suffered from cancer within the clinical spectrum of the *BRCA1*-associated tumors. However, the carrier status of the proband’s brother confirms that the pathogenic variant was already present in the germline lineage of one of their parents. While purely speculative, this raises the remote possibility that the insertion arose as a de novo event in one of the parents. This aligns with the observation that, although most processed pseudogenes in the human genome accumulate numerous sequence variants relative to their active counterparts [28], only three minor base alterations were detected in the studied insertion—one of which is indicative of the non-homologous end joining (NHEJ) mechanism responsible for sealing DNA strands [29]. The modest sequence divergence from the host gene indicates that this transposition may have happened recently.

## 5. Conclusions

Discovery of the genetic causative factors and pathological interpretation of the genotyped findings are a constant problematic issue for diagnostic laboratories and for genetic counselling. Our comprehensive molecular genetic study [30] includes periodic reannotation of all variants—especially those falling in the clinically unknown category at the time of disclosure—according to the latest clinical findings, regularly following novel ACMG locus-specific guideline recommendations. We constantly keep pace with the state-of-the-art techniques and re-sequence highlighted cases with novel or more robust techniques to discover missed pathogenic variants. Where available and needed, we perform RNA-level functional studies, and we collect all relevant individual and familiar cancer data in order to assess the segregation of the variant and the phenotype.

In conclusion, we report a heritable processed transcript insertion—a previously undescribed pathogenic mechanism contributing to the development of a novel pathogenic *BRCA1* variant. To our knowledge, this is the first documented example of such an event in the literature. Importantly, conventional germline diagnostic workflows, which typically do not include structural variant (SV) analysis, would likely fail to detect this alteration. The identification required a dedicated and technically challenging analytical approach, underscoring the need to integrate comprehensive SV detection into routine testing pipelines. Our findings highlight the clinical relevance of this mechanism, as its recognition is essential for correctly identifying individuals with hereditary cancer predisposition and for enabling appropriate genetic counselling, surveillance, and risk-reducing strategies.

## Data Availability

The data presented in this study are available on request from the corresponding author.

## Supplementary Materials

The following supporting information can be downloaded at: https://www.mdpi.com/article/10.3390/cancers17233872/s1, Figure S1: Insertion detection in different tissue specimens of the patient; Figure S2: Original agarose gels for Figure 1B; Figure S3: Original agarose gels for Figure 1B; Figure S4: Original agarose gels for Figure 2B; Figure S5: Original agarose gels for Figure 2D; Table S1: Primers used for molecular techniques.

## Abbreviations

The following abbreviations are used in this manuscript:

MLPA	Multiplex Ligation-based Probe Amplification
HBOC	Hereditary Breast-and Ovarian Cancer
NGS	Next-Generation Sequencing
PARP	Poly (ADP-ribose) polymerase
SINE	Short Interspersed Nuclear Elements
