# Supplementary material for: Processed Transcript Insertion as a Novel Germline Mutational Mechanism in BRCA1-Associated Hereditary Breast Cancer
Source: Cancers (Basel). 2025 Dec 2;17(23):3872. doi: 10.3390/cancers17233872 (PMC12691391; doi:10.3390/cancers17233872)
Supplement: Supplementary file 1 [file cancers-17-03872-s001.zip › cancers-3995506-supplementary.pdf]

cancers

MDPI

Supplementary material: Processed Transcript Insertion as a Novel Germline Mutational Mechanism in *BRCA1*-Associated Hereditary Breast Cancer

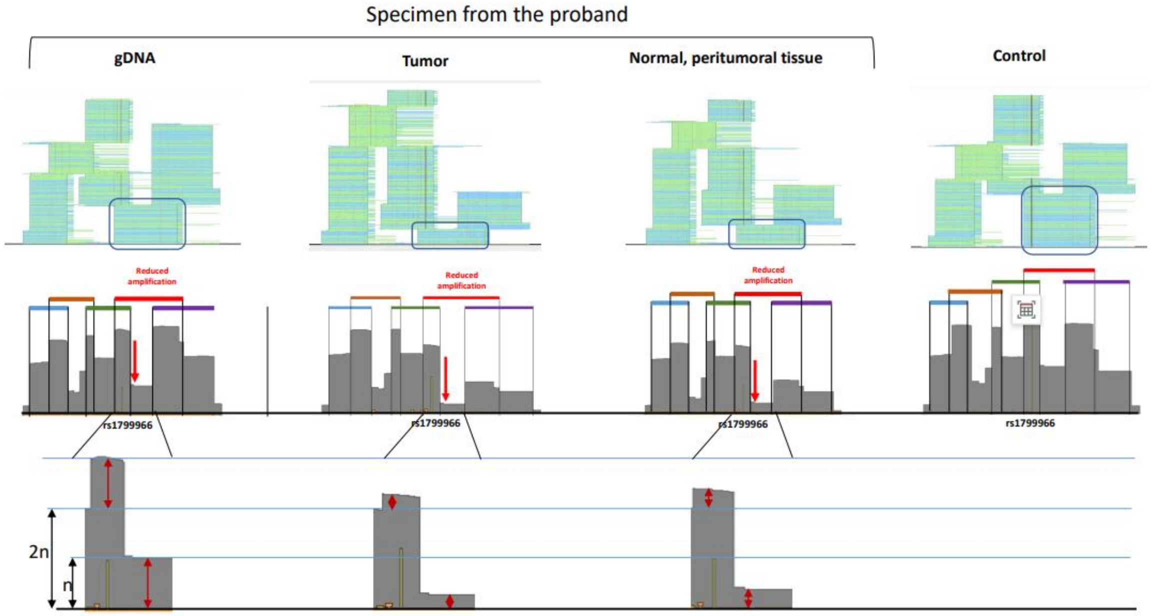

Figure S1: Insertion detection in different tissue specimen of the patient

Library was prepared by amplicon-based method for BRCA1/2 genes (#A32840 Oncomine™ BRCA Research Assay, Illumina) from somatic DNA of surgical specimen as well as blood-derived genomic DNA of the proband and sequenced with Ion GeneStudio™ S5 Plus SystemNGS sequencer (ThermoFisher Scientific, MA, USA). Middle row: Read coverages of BRCA1 exon 16 region. Cumulative read coverages from .bam files are visualized in Golden Helix Genome Browser (v.3.0) and exon 16 region is imaged by screenshot. Library-generation amplicons are depicted with coloured lines above the coverage bars. Red arrow signifies the insertion point. Genotyped rs1799966 variant is detectable as yellow bar. Control is from germline DNA of a normal (ref. seq.) sample. The coverage of the amplicon overlapping the insertion (with red) was obviously smaller in the specimens of the proband than the control sample, relative to nonoverlapping amplicons (i.e. amplicon with green). Reduced amplification: the overlapping (red) amplicon is not amplified from the insertion-carrying allele. Exact quantitaive calculations addressing insert-carrying vs normal allele ratios were not amenable based on read coverages, because of the considerable standard deviation of this metric inherent to amplicon-based enrichment methods. Upper row: Pile-up representation of the reads All reads generated from the amplicons are depicted beneath the proper regions. Forward and reverse reads are coloured with blue and green respectively. rs1799966 variant was genotyped heterozygote in all specimen of the proband and homozygote in the control. The reads, coming from insert-containing amplicon (red) of the patient did not contain rs1799966, maintaining the observation that alternative allele of this marker was in phase with the insertion, therefore not amplified. Note the significant difference in relative amount of the reads of this region (highlighted with frame). Patient samples yielded much less reads of the framed region than control, signifying that non-amplified insertion-carrying allele was present in each of the patient's specimen. Bottom row: Marker-based calculations of allele ratios Harnessing the genotyping results of the heterozygote marker rs1799966, we could make a hint for insert-carrying vs normal allele ratios of the patient specimen. Regions

covering two amplicons (red and green) are highlighted of each figure of the middle row. Subtracting the reads produced from the red region (red doubleheaded arrow) from the green one we resulted the net reads generated exclusively from the green amplicon. The ratio of the heterozygote marker rs1799966 (with yellow bar) reflected well the insertion content. In all DNA specimen of the patient it was 1:1, except for the tumor. In tumor, alternative allele of the marker prevailed, signing a shift towards insert-carrier allele.

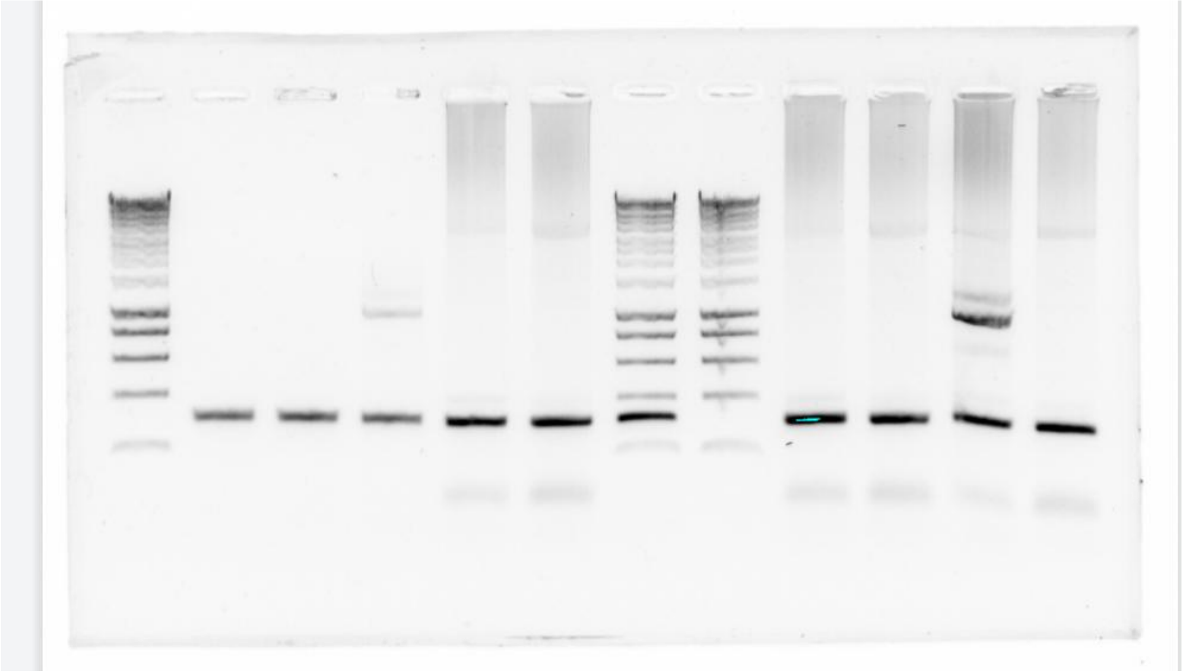

Figure S2: Original agarose gels for Figure 1B

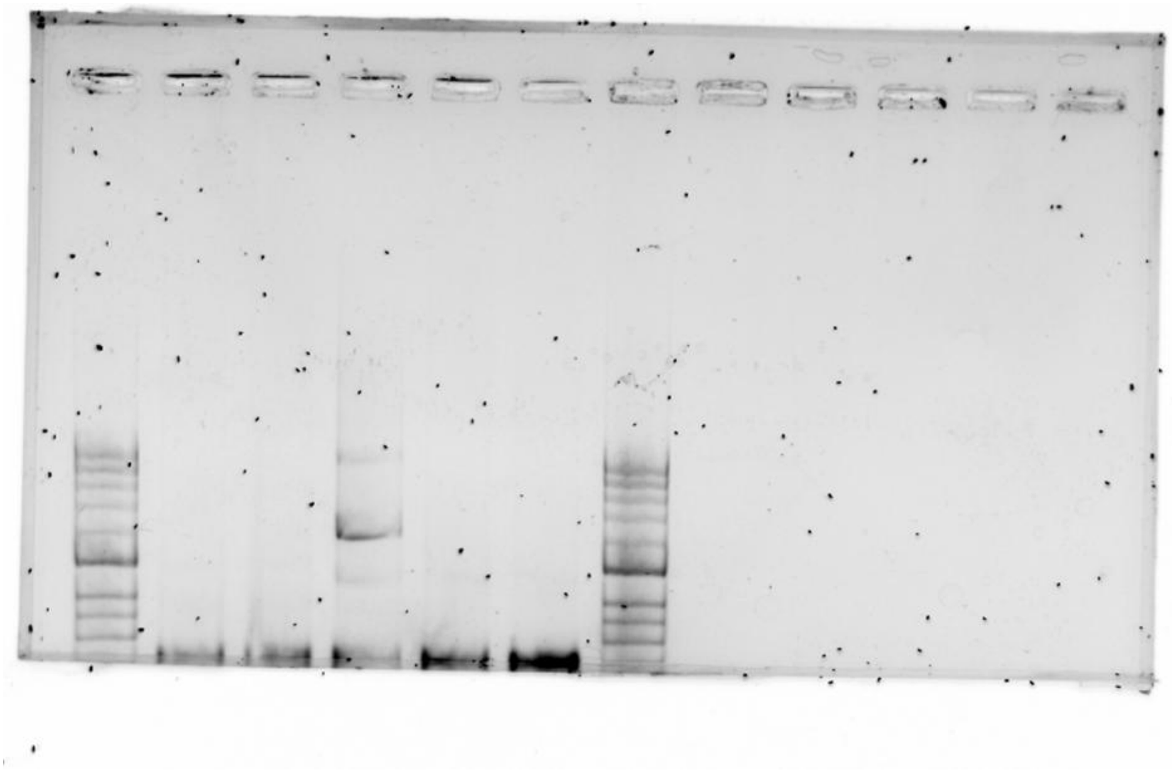

Figure S3: Original agarose gels for Figure 1B

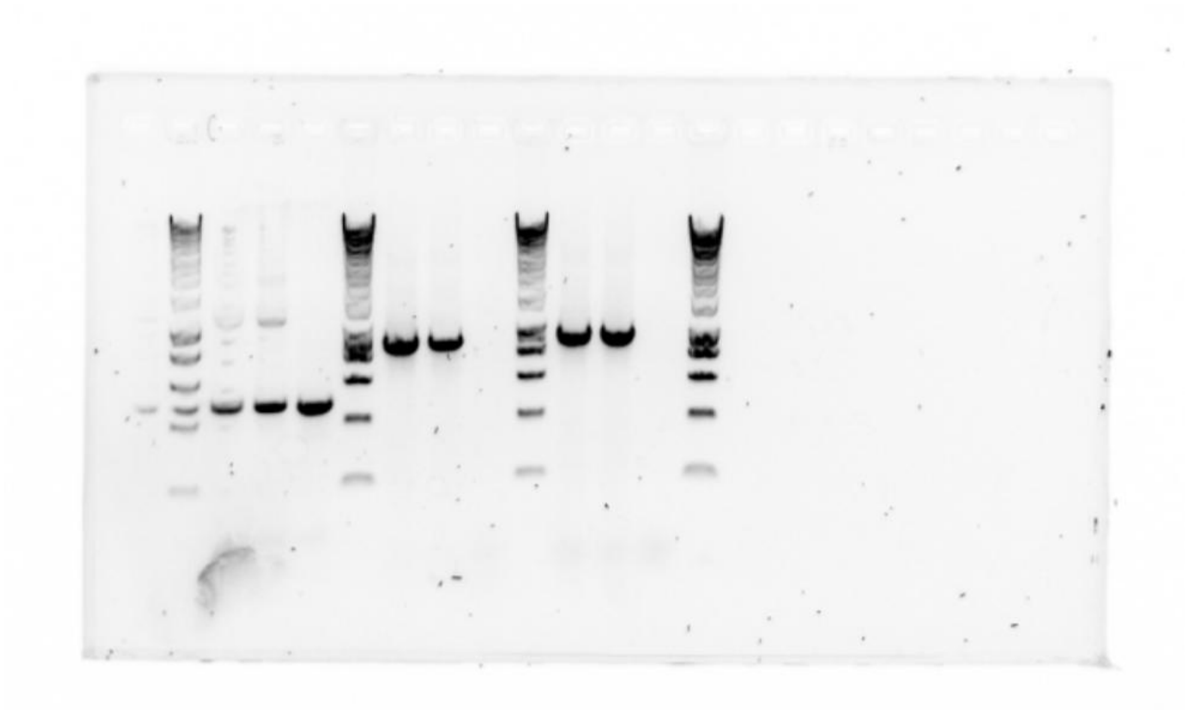

Figure S4: Original agarose gels for Figure 2B

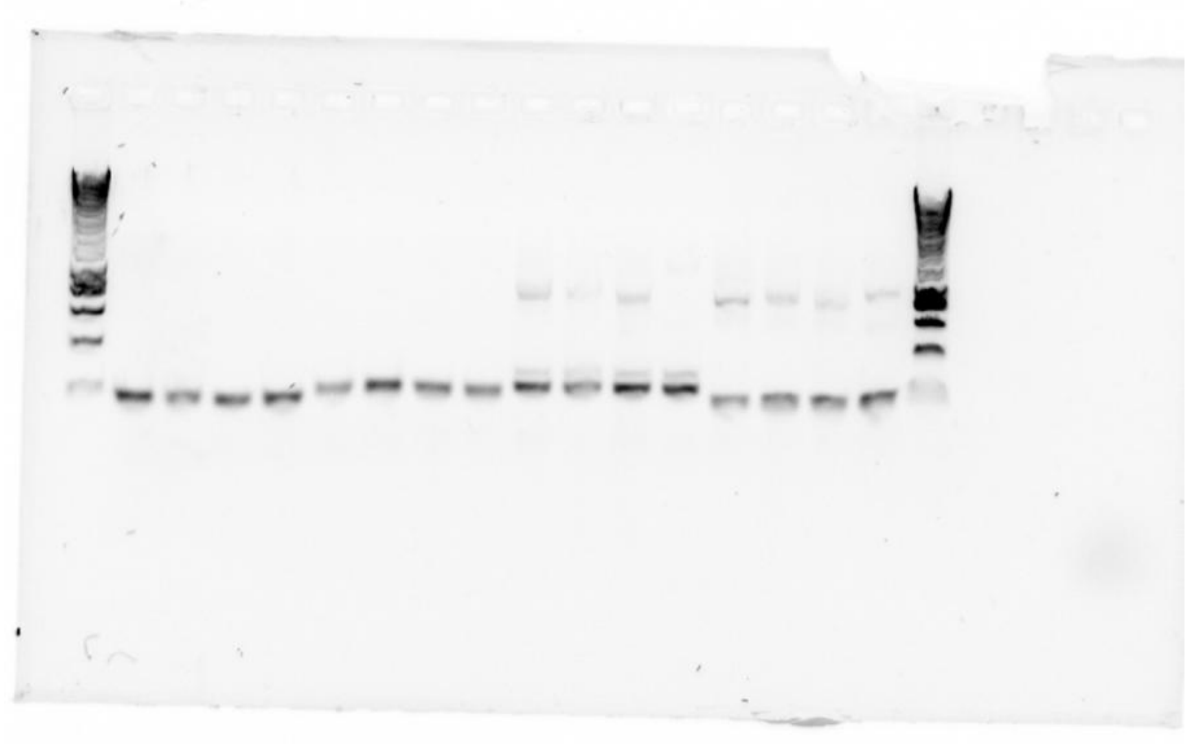

Figure S5: Original agarose gels for Figure 2D

Supplementary Table S1: Primers used for molecular techniques

**Genomic DNA PCR primers:**		
BRCA1_ex15_F	GCC AGT CAT TTC TGA TCT CTC TG	Long-range PCR
BRCA1_ex17_R	GCC TCA TGT GGT TTT ATG CA	Long-range PCR
BRCA1_ex16_F	ACA GAG ACC AGA ACT TTG TAA TTC AA	Nested sequencing from long-range PCR
BRCA1_ex16_R	CCT CTA GGT TAT TAA TTG ACA ATA CCT ACA T	Nested sequencing from long-range PCR

B1_ex16_ins_tp_F	AAA GTT CCC CAA TTG AGC TTT T	Nested sequencing from long-range PCR
B1_ex16_ins_F	GGC ACG CTA CGA GAG TAC AA	Nested sequencing from long-range PCR
**Tumor DNA PCR primers:**		
BRCA1_ex16_FOR	TGA AGA CAG AGC CCC AGA GT	
InsRPL18_REV	CAG CGA CCC ACT ACC TTG TA	designed insisde the insert
BRCA1_ex16_REV	CCC TGC TCA CAC TTT CTT CC	
InsRPL18_FOR	GCC CAA TAA ACT CAG GAA CG	designed insisde the insert
**cDNA primers for RT-PCR:**		
Detection of the insert:		
BRCA1_ex16_FOR	TGA AGA CAG AGC CCC AGA GT	inside exon 16, flanking the insert
BRCA1_ex16_REV	CCC TGC TCA CAC TTT CTT CC	inside exon 16, flanking the insert
Allele imbalance primers:		
B1_C_ex16_FOR	GAA AGT TCC CCA ATT GAA AGT T	designed for unbiased amplification of rs1799966
BRCA1_ex17_R	GCC TCA TGT GGT TTT ATG CA	designed for unbiased amplification of rs1799966
Real-time PCR primers:		
BRCA1_ex16_FOR	TGA AGA CAG AGC CCC AGA GT	specific to aberrant transzkript (PCR 1)
InsRPL18_REV	CAG CGA CCC ACT ACC TTG TA	specific to aberrant transzkript (PCR 1)
InsRPL18_FOR	GCC CAA TAA ACT CAG GAA CG	specific to aberrant transzkript (PCR 2)
BRCA1_ex16_REV	CCC TGC TCA CAC TTT CTT CC	specific to aberrant transzkript (PCR 2)
B1_C_ex16_FOR	GAA AGT TCC CCA ATT GAA AGT T	designed for target site duplication, ampifying both aberrant and normal transcripts (PCR3)
BRCA1_ex17_cDNS_Rev	TGG CAA ACT TGT ACA CGA GCA	ampify both aberrant and normal transcripts (PCR3)
